# Maternal and neonatal outcomes in pregnant women with autoimmune diseases in Pavia, Italy

**DOI:** 10.1186/s12887-015-0532-3

**Published:** 2015-12-18

**Authors:** Iolanda Mazzucchelli, Lidia Decembrino, Francesca Garofoli, Giulia Ruffinazzi, Véronique Ramoni, Mariaeva Romano, Elena Prisco, Elena Locatelli, Chiara Cavagnoli, Margherita Simonetta, Annalisa De Silvestri, Piermichele Paolillo, Arsenio Spinillo, Mauro Stronati

**Affiliations:** Neonatal Unit and Neonatal Intensive Care Unit, IRCCS San Matteo Hospital Foundation, Pavia, Italy; Department of Internal Medicine and Therapeutics, University of Pavia, Pavia, Italy; Division of Rheumatology, IRCCS San Matteo Hospital Foundation, University of Pavia, Pavia, Italy; Department of Obstetrics and Gynecology, IRCCS San Matteo Hospital Foundation, University of Pavia, Pavia, Italy; Biometry Unit, San Matteo Hospital Foundation, P.le Golgi 19, Pavia, 27100 Italy; Division of Neonatology and Neonatal Intensive Care, Casilino General Hospital, Roma, Italy

**Keywords:** Autoimmune disease, Pregnancy, Maternal and neonatal outcome

## Abstract

**Background:**

The increased number of childbearing women with autoimmune diseases leads to a growing interest in studying relationship among maternal disease, therapy, pregnancy and off-spring. The aim of this study was to determine the impact of autoimmune disease on pregnancy and on neonatal outcome, taking into account the maternal treatment and the transplacental autoantibodies passage.

**Methods:**

We studied 70 infants born to 70 pregnant women with autoimmune disease attended in Fondazione IRCCS Policlinico San Matteo, Pavia, Italy from June 2005 to June 2012. Maternal and neonatal characteristics were collected and relevant clinical, laboratory, therapeutics, sonographic and electrocardiographic investigations were recorded and analyzed.

**Results:**

We observed a high rate of spontaneous abortions in medical history, 29 %, and 18.6 % of preterm births and 22.9 % of low birth weight (< 2500 g). Transplacental autoantibodies passage wasn’t related to maternal or obstetrical complication, but anti-Ro/SSA positive pregnancies correlated with abnormal fetal heart rate (*P* = 0.01). Pregnant women on therapy showed an higher incidence of maternal (*p* = 0.002), obstetric (*p* = 0.007) complications and an increased rate of intrauterine growth restriction (*p* = 0.01) than the untreated ones.

**Conclusions:**

Autoimmune diseases in pregnancy require to be carefully monitored to ensure the best possible management of mothers, fetuses and newborns due to the high rate of morbidity specially in case of maternal polytherapy and/or anti-Ro/SSA positivity.

## Background

Autoimmune diseases are a group of heterogeneous disorders characterized by the attack of the immune system against its own cells, tissue or organs. In this context the resultant autoinflammatory reaction could be antibodies mediated or cytokines/protein mediated, with consequently multiple clinical and laboratory aspects [1]. Diseases are more frequent in female gender than in male (78 %) and they could become critical during pregnancy when the immune system is actively involved to tolerate a successful gestation process [2, 3]. A pregnant woman with an autoimmune disease, should be considered high risk because of the modulation of inflammatory process, the possible effects of therapy on the fetus and the transplacental passage of autoantibodies [4, 5]. High frequency of gestational and perinatal complications such as preeclampsia, stillbirth, spontaneous abortions (11–24 %), preterm birth (13.9 %) and intrauterine growth restrictions (IUGR) (2.3 %) have been reported [6, 7]. Immunoglobulin G isotype antibodies can freely cross the placenta with a linear relationship between gestational age and placental transfer [5]. Literature data report that anti-Ro/SSA and anti-La/SSB antibodies are associated with neonatal lupus erythematosus and other associated abnormalities such as congenital heart block (CHB), cardiomyopathy, cutaneous lupus lesions, hepatobiliary disease, and hematologic cytopenias [8, 9]. Furthermore, an important issue concerns the use of drugs to control maternal symptoms and exacerbations: these substances and their metabolites can cross the placental barrier and enter into the fetal circulation; this could influence the pregnancy progression, the fetus state and the perinatal outcomes [4].

The aim of this study was to expose our experience about the impact of autoimmune diseases on pregnancy, fetus and neonate outcome, taking into account the transplacental autoantibodies passage and the maternal therapy.

## Methods

This retrospective study was performed at Fondazione IRCCS Policlinico San Matteo, Pavia, Italy from June 2005 to June 2012 and data of 70 women with a diagnosed autoimmune disease and of their 70 babies were collected and analyzed. The hospital, located in the north of Italy, is characterized by clinical, teaching and research mission. All the women gave written informed consent and the data was collected in a database and analyzed as anonymous data for research purpose.

The study was approved by the Ethics Committee, IRCCS Fondazione Policlinico San Matteo, Pavia, Italy.

All the enrolled women had a diagnosed autoimmune diseases in our Division of Rheumatology according to widely used criteria [10] and had a positive test for at least one autoantibody. Exclusion criteria were: multiple pregnancy, congenital infections, perinatal asphyxia, chromosomal syndromes. Any abnormalities occurring during pregnancy were registered as well as obstetrical and maternal information. Blood tests to evaluate maternal autoantibodies were performed during pregnancy according to the clinical protocol used in our Department.

Neonatal gestational age, weight, length, head circumference, Apgar score and cord pH were collected at birth and adjusted for gestational and chronological age. Maternal and neonatal antinuclear autoantibodies (ANA) were tested by a standard indirect immunofluorescence technique, while anti-extractable nuclear antigen antibodies (ENA) were evaluated by commercially available ELISA kits. Cardiac and cerebral ultrasound scans were performed in the neonate at birth, while electrocardiogram (ECG) testing was done within the first week of life. Electrocardiographic parameters were evaluated with particular attention to PR interval (nv <140 ms), heart rate, QT interval corrected according to the Bazett’s formula (QTc) (nv <440 ms): slightly extended if the value is in the range of 440–470 ms and frankly abnormal if > 470 ms.

### Statistical analysis

The Shapiro–Wilk test was used to test univariate normality of quantitative variables. When these were normally distributed, results were expressed as mean values and SD, otherwise median values and interquartile range were used (IQR; 25–75^th^ percentile). Data were analyzed adequately by Student *t* test or Mann–Whitney and by analysis of variance (ANOVA) or Kruskal-Wallis test if more than two groups were involved. All tests were two-sided. *P* values ≤ 0.05 were considered statistically significant. Association between quantitative variables was evaluated by Spearman non parametric correlation. Qualitative variables were described by counts and percentages and data were analyzed by Fisher’s exact test. Data analysis was performed using STATA statistical package (version 12; Stata Corporation, College Station, 2011, Texas, USA).

## Results

Characteristics of the enrolled women are reported in Table 1. Obstetrical history of 70 women revealed an incidence of 29 % of miscarriages (40 out of 136 pregnancies).

**Table 1:** Characteristics of pregnant women

Mothers, number	70
Age, mean (SD)	33.3 years (5.1)
Pregnancy, number (%)	
Term delivery (≥37 weeks)	57 (81.4)
Preterm delivery (<37 weeks)	13 (18.6)
Mode of delivery, number (%)	
Vaginal delivery	33 (47.1)
Caesarean section	37 (52.9)
Autoimmune diagnosed disease, number (%):	
Sjögren’s syndrome (SS)	8 (11.4)
Systemic lupus erithematosus (SLE)	9 (12.9)
Rheumatoid arthritis (RA)	3 (4.3)
Antiphospholipid syndrome (PAPS)	5 (7.1)
Undifferentiated connective tissue disease (UCTD)	19 (27.1)
Others^a^	8 (11.4)
Not clinical manifest disease^b^	18 (25.7)
Drug therapy during pregnancy, number (%)	37 (52.9)

^a^ *n* = 1 autoimmune thyroiditis, *n* = 4 thrombocytopenia, *n* = 1 thyroiditis and autoimmune gastritis, *n* = 1 ulcerative colitis, *n* = 1 Wegener’s granulomatosis. All were ANA positive

^b^Women positive to autoantibodies without clinical features of autoimmune disease

Maternal complications observed among the 70 enrolled pregnant women were observed in 13 patients (19 %): 2 cases of gestational diabetes, 5 of hypertension, 1 of massive pulmonary embolism, 1 of hypertransaminasemia and hyperbilirubinemia, 1 of anemia and furthermore, 1 autoimmune uveitis, 1 onset of Systemic lupus erithematosus (SLE), 1 intrahepatic cholestasis due to the worsening of the autoimmune disease.

Obstetric complications observed among the 70 enrolled pregnant women were observed in 29 cases (41.4 %): abnormal fetal heart rate *n* = 7 (10 %), meconium stained amniotic fluid *n* = 8 (10.7 %), oligohydramnios *n* = 3 (4.3 %), polyhydramnios *n* = 2 (2.9 %), threat of preterm delivery *n* = 3 (4.3 %), IUGR *n* = 4 (5,71 %), positive indirect Coombs test *n* = 2 (2.9 %).

Neonatal characteristics are reported in Table 2.

**Table 2:** Characteristics of infants enrolled in the study

Gestational age, mean (SD)	38 weeks (3)
Sex, number (%)	
Male	35 (50.0)
Female	35 (50.0)
Apgar score at 1′ and 5′, mean (SD)	9.0 (1.5) and 9.7 (0.9)
Cord pH, mean (SD)	7.27 (0.10)
Blood parameters,	
White Blood Cells x10^3^/μl, mean (± SD)	15.3 (5.9)
Neutrophils x10^3^/μl, mean (± SD)	8.6 (4.5)
Hemoglobin g/dL, mean (± SD)	17.2 (2.0)
Hematocrit, %	51.6 (6.5)
Platelets x10^3^/μl, mean (± SD)	270.9 (75.5)
Anthropometric data,	
Weight at birth, mean (SD)	3045 gr (699)
LBW^a^ number (%)	14 (20.0)
VLBW^b^ number (%)	1 (1.4)
ELBW^c^ number (%)	1 (1.4)
Length, mean (SD)	49 cm (3.5)
Head circumference, mean (SD)	34 cm (2.4)
IUGR^d^, number (%)	4 (5.7)

^a^Low Body Weight (Birth weight < 2500 g)

^b^Very Low Body Weight (Birth weight < 1500 g)

^c^Extremely Low Body Weight (Birth weight < 1000 g)

^d^IUGR Intrauterine growth restriction

All the newborns were tested for maternal autoantibodies at birth to assess the transplacental passage (Table 3) and we didn’t observe significant differences comparing data obtained from infants positive to maternal autoantibodies (46 out of 70) vs those that were negative (18 out of 70). No differences in obstetric and perinatal complications (Table 4), mode of delivery, gestational age, anthropometric parameters at birth and laboratory data, except for total bilirubin levels (7.2 vs 9.8 mg/dL, *p* = 0.05) were observed between infants positive to maternal autoantibodies and those negative. In regard to the tested electrocardiographic values, no differences were found in the two groups. We observed that Ro/SSA and La/SSB autoantibodies crossed the placental barrier in 100 % of cases (Table 3) and five out seven neonates with abnormal fetal heart rate were born to mothers positive for anti-Ro/SSA (*p* = 0.01), of these five neonates one was born to mothers treated with hydroxychloroquine, one to mother with steroid and three to mothers without therapy. Moreover, the routine analysis of neonatal laboratory parameters of infants born to mothers positive to anti-Ro/SSA versus infants born to negative ones, didn’t show any statistically difference. We noted higher hemoglobin (*p* = 0.007), higher hematocrit (*p* = 0.02) and higher total bilirubin levels (*p* = 0.008) in the off-spring of negative mothers to anti-Ro/SSA, all these values were however in normal ranges.

**Table 3:** Autoantibodies detected in mothers and in the newborns at birth

Autoantibodies	Mothers (*n* = 70)	Infants (*n* = 70)	Placental transfer (%)
ANA	63	46	73
anti-SSA/Ro	22	22	100
anti-SSB/La	6	6	100
Others^a^	3	0	0

^a^other autoantibodies

**Table 4:** Obstetric and perinatal events compared to autoantibodies transplacental transfer

Obstetrical/perinatal events	Autoantibodies placental transfer		
	Yes (*n* = 46)	No (*n* = 18)	*P*-value
Abnormal fetal heart rate	6	1	NS
Meconium stained amniotic fluid	5	3	NS
Oligohydramnios	1	2	NS
Polyhydramnios	2	0	NS
Threat of preterm delivery	1	2	NS
IUGR	3	1	NS
Positive indirect Coombs test	2	0	NS
Ventricular septal defect	1	0	NS
Cerebral alteration	4	4	NS

*NS* Not-Significant

Moreover, maternal and neonatal ANA titre were highly correlated as evaluated by the Spearman rho 0.74, *p* < 0.001.

Thirty-seven (52.0 %) pregnant women received medical therapy, they were treated with one or more drugs for their autoimmune disease: 23 received steroid therapy (prednisone), 9 received hydroxychloroquine, 17 received low dose aspirin and 7 low molecular weight heparin. Statistical analysis showed a higher incidence of obstetrical (*p* = 0.007) and maternal (*p* = 0.002) complications in treated women than in non-treated ones, related to a higher incidence of preterm delivery (*p* = 0.06) with low birth anthropometric parameters: weight (*p* = 0.003), length (*p* = 0.01) and head circumference (*p* = 0.004). In women undergoing both immunosuppressive and anti-coagulant/anti-platelet therapy a significant correlation with a higher incidence of IUGR newborns compared to untreated women (*p* = 0.01) was registered.

Echocardiography revealed one case of ventricular septal defect in a IUGR preterm neonate.

Cerebral ultrasound scans revealed in 8 newborns the following significant alterations: periventricular hyperechogenicity (*n* = 6), intraventricular hemorrhage which resulted in pseudocysts or in multicystic leukomalacia (*n* = 4), white matter leukodystrophy associated with agenesis of the corpus callosum (*n* = 2). The statistical analysis showed that these cases correlate significantly with mother’s drug therapy, *p* = 0.005, but no correlation was observed with the presence of anti-Ro/SSA, *p* = 0.4.

## Discussion

Our observations, in agreement with data reported by other authors, confirmed a higher frequency of miscarriages, of premature births and of low birth weight infants born to women with autoimmune disease compared to epidemiologic data of general obstetric population in developed countries [11]. Moreover, we confirmed previous literature data showing that the placental barrier is crossed by antibodies: more than 70 % of ANA and 100 % of anti-Ro/SSA and anti-La/SSB [12, 13].

The association of anti-Ro/SSA maternal positivity with neonatal lupus complicated by the congenital sinus bradycardia, first degree AV block or elongated QTc has been reported [14]. Our findings, instead, did not reveal any neonatal lupus disease in the off-spring from positive mothers. Nevertheless, a statistically significant correlation between anti-Ro/SSA positive pregnancies and abnormal fetal heart rate at the tococardiographic tracing was detected (*p* = 0.01). The arrhythmogenic role of anti-Ro/SSA and anti-La/SSB was established in animal and in human [13, 15] this effect could promote CHB in newborns of mothers Ro/SSA and/or La/SSA positive. We therefore supposed that the CHB has possibly been prevented by an adequate and carefully monitored pharmacological treatment during pregnancy. In our study, in fact, 73 % of the mothers anti-Ro/SSA positive received a pharmacological treatment to control their autoimmune disease.

To date, even if there is no full agreement about the effects of immunosuppressive drugs on pregnancy and on fetus/infant, high-dose of immunosuppressive treatment administered to transplanted pregnant women has been associated with a transient impairment of the immune system of the newborn, increasing the risk of neonatal infections in the first months of life [16]. Other authors carried out a study to evaluate relevant effects in newborns and children born to women receiving immunosuppressants for their autoimmune disorders. No relevant effect on neonatal immune function, as evaluated by complete blood count with differential white blood cell count, immunoglobulin concentrations, lymphocyte subpopulations, and later in response to hepatitis B vaccination were revealed [17]. In our study, as well, neither neutropenia nor difference between white blood cells and neutrophils count were reported in infants born to treated women, compared to those born to untreated mothers.

Furthermore, our findings revealed a statistical significant higher incidence of obstetric and maternal complication in treated women compared to those untreated. Previously, an increased risk of preterm delivery, low birth weight and IUGR associated with immunosuppressant therapy was reported by other authors [18].

We observed significantly lower values of neonatal anthropometric parameters at birth (weight, height and head circumference) in the infants born to treated mothers compared to untreated ones. Nevertheless we have to remark that these infants had an earlier gestational age at birth too, which may be the reason of the lower anthropometric measures. Interestingly, a higher number of IUGR newborns (*p* = 0.01) was observed in the group of women receiving polytherapy than in the group of the untreated women. Unfortunately, we aren’t able to conclusively address the correlation of this event either with the polytherapy or with the worsening disease, requiring itself a more important therapy to control increasing symptoms. In fact both the conditions coexist and are strictly linked.

Finally, the ultrasound evaluation showed cerebral alterations in 8 infants born to treated mothers, but these alterations didn’t correlate to the mother’s positivity to anti-Ro/SSA. Because the use of the drugs may be an indirect sign of the disease, it is unclear if the alterations may be related to drug therapy, to the autoimmune disease or the results of the add up of these factors. These data comply with the observations of Motta et al. [19] that suggested no specific perinatal risk factor, but a multi-factorial aetiology of brain abnormalities observed in infants born to mothers with autoimmune disease.

A recent published review highlighted that the principal immunomodulant/immunosuppressive drugs administered during pregnancy have been shown to be relatively safe, but the results are not conclusive [20]. Moreover, controlled clinical trials in pregnancy are not ethical, therefore monitoring the pregnancy of these patients is the only available tool to have safety assessment. In agreement with the cited authors we also hope that international registries of the potential drug side effects or of any useful information related to the pregnancy and the outcome of the off-spring will become common and easily accessible over different countries [21]. Furthermore, it must be considered that autoimmune diseases could be undiagnosed before pregnancy because the mild clinical illness that could be modified by the occurring pregnancy that interferes with the immune system. The administration of a screening questionnaire in the first trimester of gestation has shown to be a useful tool to identify in advance these subjects that could be monitored carefully and, if necessary, managed the appropriate therapy to proceed successfully with the pregnancy [22].

We have therefore to take care of this dichotomous aspect: monitoring and balancing the necessity to identify and treat maternal disease and to preserve fetal health. In fact, it is important to protect mother and fetus from the hazard of increasing symptoms without, on the other hand, compromising the newborn well-being with possible drug side effects.

We are aware of the limits of our study. The sample size didn’t allow to create subgroups related to different autoimmune diseases. Furthermore, we could not state if the findings were related to maternal disease or to drugs therapy or to others causative factors, such as genetic features or neonatal independent circumstance (prematurity), because some of these conditions are very often concomitant. Finally, we had not the opportunity to follow the infants’ growth, therefore we have no information about the possible sequelae afterward in childhood.

## Conclusion

The take home message of this investigation is that pregnancy of mothers suffering for an autoimmune disease has to be carefully monitored. Placental transfer of maternal autoantibodies, and the possible effects of maternal therapy on the fetus/neonate are risk factors that require a careful multidisciplinary management (gynecologists, rheumatologists and neonatologists) to ensure the best possible maternal and neonatal outcome International registries describing the pattern of pregnancy and the outcome of the off-spring could be an useful tool to ameliorate the management of the mother/infant dyad.

## Abbreviations

ANA: Antinuclear autoantibodies
CHB: Congenital heart block
ECG: Electrocardiogram
ENA: Anti-extractable nuclear antigen antibodies
IQR: Interquartile range
IUGR: Intrauterine growth restrictions
SLE: Systemic lupus erithematosus
